# Supplementary material for: Content-rich biological network constructed by mining PubMed abstracts
Source: BMC Bioinformatics. 2004 Oct 8;5:147. doi: 10.1186/1471-2105-5-147 (PMC528731; doi:10.1186/1471-2105-5-147)
Supplement: Additional File 2 — The original results of the above study (non-essential files are deleted to keep the file size under the limit set by BMC bioinformatics). [file 1471-2105-5-147-S2.bz2 › chilibotAdditionalFile2/dip05/174ID9461436E589/html/E4TF1A_GABPB1.html]

 


 **E4TF1A** and **GABPB1** 
  
Found 8 abstracts in PubMed, retrieved 05.  
 

 What does Google say? 
 PDF only 
| .edu only 

---

**Interactive relationship** (e.g. stimulation, inhibition, etc)

**Non-interactive relationship** (e.g. studied together, co-existance, homology, etc.)

- Furthermore, coexpression of E4TF1  [ **GABPB1** ]  60  [ **E4TF1A** ]  and E4TF1 53  [ **GABPB1** ]  markedly increased aurora A promoter activity.  Ref: 11790771 J Biol Chem, 2002
- Mapping of the human transcription factor GABPA E4TF1  [ **GABPB1** ]  60  [ **E4TF1A** ]  gene to chromosome 21.  Ref: 7590737 Genomics, 1995
- The gene encoding human transcription factor E4TF1  [ **GABPB1** ]  60  [ **E4TF1A** ]  was previously mapped to chromosome 21q21.  Ref: 8543189 Gene, 1995
- Furthermore, co expression of E4TF1  [ **GABPB1** ]  60  [ **E4TF1A** ]  and E4TF1 53  [ **GABPB1** ]  47 squelched TPO gene expression in PLC and HepG2 cells.  Ref: 9111044 J Biol Chem, 1997
- We analyzed the localization of the E4TF1  [ **GABPB1** ]  60  [ **E4TF1A** ]  gene in more detail by genomic Southern hybridization and determined the sequence of the exons and the regions surrounding the intron boundaries.  Ref: 8543189 Gene, 1995
- In addition to APP, the cosmid contains the E4TF1  [ **GABPB1** ]  60  [ **E4TF1A** ]  transcription factor, the U2AF35 pre mRNA splicing factor, and the cystathionine beta synthetase CBS gene.  Ref: 9931491 Gene, 1999
- Anti E4TF1  [ **GABPB1** ]  60  [ **E4TF1A** ]  GABPalpha or anti E4TF1 53  [ **GABPB1** ]  47 GABPbeta antibodies supershifted the complex in gel shift assay.  Ref: 9111044 J Biol Chem, 1997
- Assignment of the E4TF1  [ **GABPB1** ]  60  [ **E4TF1A** ]  gene to human chromosome 21q21.2 q21.3.  Ref: 8543189 Gene, 1995
- We report here that E4TF1  [ **GABPB1** ]  60  [ **E4TF1A** ]  locates in the long arm of chromosome 21 at q21.2 q21.3 and contains a total of ten exons.  Ref: 8543189 Gene, 1995
- The introns of APP and E4TF1  [ **GABPB1** ]  60  [ **E4TF1A** ]  are 49 and 24 fold smaller in Fugu than in human, and the intergenic distance is compressed at least 100 fold.  Ref: 9931491 Gene, 1999
- Anti E4TF1  [ **GABPB1** ]  60  [ **E4TF1A** ]  antibody generated a supershifted complex.  Ref: 11790771 J Biol Chem, 2002
- APP and E4TF1  [ **GABPB1** ]  60  [ **E4TF1A** ]  map within 21q21.  Ref: 9931491 Gene, 1999
- One trapped sequence showed complete homology with nucleotide sequence D13318 of GenBank, which corresponds to the gene for human transcription factor E4TF1  [ **GABPB1** ]  60  [ **E4TF1A** ]  HGMW approved nomenclature GABPA.  Ref: 7590737 Genomics, 1995
